# Drugs, Microbes, and Antimicrobial Resistance

**DOI:** 10.3201/eid1106.AC1106

**Published:** 2005-06

**Authors:** Polyxeni Potter, comments to authors

**Affiliations:** * Centers for Disease Control and Prevention, Atlanta, Georgia, USA

**Keywords:** Art and science, emerging infectious diseases, drugs, richard estes, antimicrobial resistance, superrealism, photorealism

**Figure:**
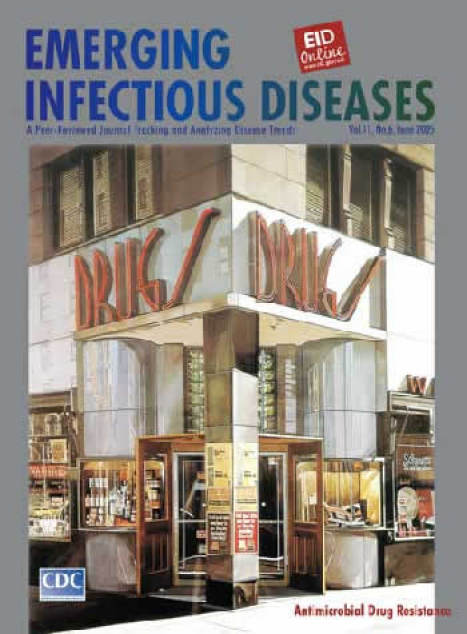
Richard Estes (b. 1932). DRUGS (1970). Oil on canvas (152.4 cm × 112.71 cm). Copyright Richard Estes, courtesy, Marlborough Gallery, New York, NY, USA

"If America is to produce great painters," wrote great American painter Thomas Eakins (1844–1916), young artists should "remain in America to peer deeper into the heart of American life" (*1*). Eakins traveled abroad, where he became familiar with the work of 19th-century greats Gustave Courbet, Édouard Manet, and Edgar Degas, but returned home to become a master of realism whose exactness and precision influenced many 20th-century American painters, one of them Richard Estes.

Estes, a native of Kewanee, Illinois, grew up in Chicago and attended the city's famed Art Institute. Upon graduation in 1956, he moved to New York to pursue a career in graphic design as freelance illustrator for magazines and advertising agencies. In the next 10 years, he crossed over to fine art and had his first exhibit around 1968 (*2*).

An admirer of Eakins, Estes peered deeply into the American cityscape for a new style of art, true not only to his native culture but also to his times. This style evoked the traditions of *trompe l'oeil* (fooling the eye through photographic illusion) and of 17th-century Dutch painting to create a contemporary version of reality (*3*). "When you look at a scene or an object you tend to scan it. Your eye travels around and over things. As your eye moves the vanishing point moves," Estes said in an interview. "…to have one vanishing point or perfect camera perspective is not realistic" (*4*).

Drawing from his surroundings rather than the imagination, Estes used photography to collect images or frozen moments of light on surfaces to complement his own recollection of places and objects. He did not reproduce photographic scenes. From multiple images, he selected certain elements, abstracting and arranging them to best advantage, exaggerating angles and omitting extraneous detail. This innovative perception and composition of visual reality came to be called superrealism or photorealism, a new art movement co-founded by Estes in the late 1960s (*5*).

Photorealists, many of them influenced by Estes, painted varied images, portraits as well as landscapes, in exquisite detail. Their subjects were diners and storefronts, gum-ball machines, neon lights, pickup trucks, and other trappings of 1970s American life. Estes was captivated by the contemporary urban landscape, particularly of New York, where he has lived much of his life, although he has also worked in Chicago, Venice, and Paris. One in a long line of artists to know and paint New York, he has worked in terms that seem architectural in their emphasis on structure and design and created of this landscape a veritable visual spectacle for posterity. Buildings, bridges, traffic patterns, city curbs were manipulated and transformed from commonplace scenes into grand theater, much more intense and "real" on canvas than ever in their own existence.

Yet, even as he has created an archivist's treasure of Downtown Manhattan and Manhattan's Upper West Side, Estes is not interested in nostalgia or future archaeologic records. And as much as he has been compared with 18th-century Italian artists (e.g., Canaletto), who painted palaces, piazzas, and canals, he is not interested in urban scenery for its beauty or underlying social commentary. He paints for the sake of painting, usually with acrylic color overlaid with oils, lovingly reinventing the scenes he explores (*6*).

"Daily life has a reputation for being banal, uninteresting, boring somehow. It strikes me that daily life is baffling, mysterious, and unfathomable" (*7*). These are the words of George Segal, Estes' contemporary and colleague, who saw magic in the mundane. Estes walks around the streets of New York until something catches his eye. He returns to the scene on weekends or evenings when the streets are deserted to take photos. Later in his studio, he reconstructs what he saw and collected, in a scene become more fiction than reality. Unlike Segal's work, which witnesses a moment of human existence, Estes' witnesses the moment itself and celebrates its visual presence with clarity and exactness.

In his meticulous reconstructions, Estes eliminates clutter, shadows, people—as if by scrubbing the scene, he can extract its essence and verify its existence. Singling out the structural, he elaborates on it from multiple angles, under a uniformly glaring light, and produces a sharp image much more compelling and deliberate than any captured by the naked eye. In the process, a perfectly dull building, an anonymous row of telephone booths, a street corner becomes arresting and memorable.

In DRUGS, on this month's cover, Estes' penetrating eye examines an icon of contemporary city life, the corner drugstore. Expertly cropped, central, and direct, the structure invites inspection on several levels: storefront and curb, window displays, and nearby buildings mirrored on shiny surfaces. A prominent column, neatly plastered with ads, blocks visual access to the interior, even with the entrance doors propped wide open against the sidewalk. The windows upstairs are shut. Elaborate glass facets and distortions of light restrict the viewer to the exterior.

Intentionally or not, Estes' drugstore, with its pristine appearance, reflects more than the block across the street. A cornerstone in the life of the city and the development of modern medicine, the institution it represents has held tricks of the medical trade, from camphor to penicillin to telithromycin. This shining apothecary symbolizes human efforts to improve health and control disease, efforts often stymied by the complexity of the task.

Not unlike artists, scientists in disease control seek order in a complicated universe. With their powerful microscopes, they too focus on the details as they construct clear, artificially uncluttered versions of a crowded microbial world. Singling out microbes that cause disease, scientists scrutinize, isolate them, and neutralize their effects on human health through powerful drugs. For their part, the microbes expel, modify, or exclude the drugs, prompting a new cycle of drug development, also destined for obsolescence. Antimicrobial drug resistance, begun with the first antimicrobial drug (*8*), threatens the single-microbe approach to disease control and the venerable institution Estes immortalized in DRUGS.
